# Relationship between calcium circulation-related factors and muscle strength in rat sciatic nerve injury model

**DOI:** 10.22038/IJBMS.2020.40915.9695

**Published:** 2020-05

**Authors:** Xiaoming Sun, Wei Wang, Yangyi Dong, Yue Wang, Meixiang Zhang, Zhao Wang, Xiaowei Yu, Jiao Huang, Hongxing Cai

**Affiliations:** 1 Department of Forensic Medicine, Xuzhou Medical University, Xuzhou, Jiangsu, P. R. China

**Keywords:** Forensic medicine, Muscle atrophy, Muscle strength, Ryanodine receptor, Sciatic nerve

## Abstract

**Objective(s):**

The purpose of this study is to investigate the indication function of the calcium circulation-related factors on the damage to muscle strength and contraction function after nerve injury. The target factors include ryanodine receptor (RyR), inositol-1,4,5-triphosphate receptor (IP3R), phospholamban (PLN), cryptocalcitonin (CASQ), ATPase and troponin C (TNNC).

**Materials and Methods:**

Sprague-Dawley (SD) rats were randomly divided into sham-operated group (SO), sciatic nerve injury group (SNI) and sciatic nerve disconnection group (SNT). Sciatic nerve function index and stretching test were used to examine the changes to muscle strength; bilateral gastrocnemius muscles were extracted after execution for gastrocnemius wet weight ratio test. HE staining slides and average cross-sectional area of muscle fibers were acquired to analyze the muscle atrophy. The transcription level of the factors was also measured.

**Results:**

Sciatic nerve damage in SNI group was significantly higher than that in SO group in the 6 weeks, but there was no significant difference between SNT and SO groups fallowing sciatic nerve damage. Sciatic nerve function in SNT group was worse than that in SNI group. The average cross-sectional area of gastrocnemius muscle fibers in SNI and SNT groups was significantly reduced compared to that in SO group. The transcriptional levels of RyR, PLN, CASQ, ATPase and TNNC in SNI and SNT groups were significantly different from those in SO group.

**Conclusion:**

Calcium circulation-related factors could be used as potential indicators for assessment of damages to muscle strength.

## Introduction

Injury of the motor system is a common type of injuries in the practice of forensic clinical identification. It is more common in sports injuries such as contusion, excessive stretching, tearing caused by accidents, muscle damage caused by surgery, etc. (1). Such injuries often impair the function of motor system, resulting in reduced muscle strength. Muscle strength refers to the strength, amplitude and speed of muscle contraction during active exercise. It is an important indicator for evaluating the body’s ability to exercise (2). The initiation and regulation of muscle strength is a complex but coordinated biological process (3) involving the direct or indirect involvement of many related genes and proteins, especially the calcium related ones. At present, the inspection methods used still rely mainly on the active cooperation of the subjects to assess their muscle strength (4,5), which leads to poor reproducibility of inspection results and lack of objective inspection indicators. Therefore, the search for indicators that can be used to objectively quantify muscle strength has become one of the urgent needs of forensic clinical identification work and clinical evaluation of muscle strength.

The basic structural unit of skeletal muscle is muscle fiber, a slender and multinuclear structure composed of multiple myoblasts. A plurality of muscle fibers surrounded by connective tissue forms a muscle bundle, and a plurality of muscle bundles forms the whole piece of muscle (6,7). 

When a pulse from the nerve reaches the skeletal muscle cells, the calcium channel on the terminal cell membrane is opened and the calcium diffuses into the sarcoplasm. Calcium ions entering the sarcoplasm bind to troponin C (TNNC), causing changes in the conformation of TNNC and detachment of actin. The myosin head then combines with actin. With the participation of ATP, the head of myosin oscillates along the long axis of the filament, which causes actin to slide along the long axis of the filament, causing myofibril shortened and resulting in muscle contraction (8). At the end of the contraction, the Ca^2+^-Mg^2+^-ATPase on the sarcoplasmic reticulum reaggregates calcium ions in the cytosol into the terminal pool (9). Ryanodine receptor (RyR), inositol 1,4,5-triphosphate receptor (IP_3_R) and phospholamban (PLN) are involved in the release of calcium ions from the sarcoplasmic reticulum; calsequestrin (CASQ) and ATPase are involved in the recovery and elimination of extracellular calcium after contraction; TNNC binds to calcium ions and participates in myofilament sliding and contraction. We investigated the transcriptional level of those calcium-related factors after nerve injury for the indication effect for muscle strength damage, to search precise quantitative indicators of muscle strength assessment for forensic clinical identification. Meanwhile, we also explored the effects of muscle atrophy on muscle strength after detecting muscle damage at the molecular level.

## Materials and Methods

***Animal model for nerve injury***

All experimental procedures were conducted in accordance with the Guidelines for Animal Experimentation of XuZhou Medical University and approved by the Committee for Animal Experimentation. One hundred 250 g male SD rats (SPF grade) were obtained from the animal center of XuZhou medical university. All rats were given free access to water and standard diet throughout the study and were maintained in a temperature-controlled (21-26 ^°^C) and humidity-controlled (50-60%) room with a 12 hr light/dark cycle. The rats were divided at random into 3 groups (n=30): sham operation (SO) group, sciatic nerve injury (SNI) group, and sciatic nerve transection (SNT) group. All experimental rats were anesthetized deeply by intraperitoneal administration of 0.2 ml/100 g 2% sodium pentobarbital. The skeletal muscles of left lower extremity were separated to expose the sciatic nerve. No further surgical operations were performed in SO group. The left sciatic nerve was crushed for 30 sec with an 18 cm forceps hemostatic in SNI group and cut off in SNT group. Five rats from each group were executed by carbon dioxide on 3 days, 1, 2, 4, 6 and 8 weeks after nerve lesion, respectively. Whole gastrocnemius muscles were excised from left and right hind limbs and measured. The ratio of the left gastrocnemius muscle weight to the right gastrocnemius muscle weight was calculated. Samples were quick-frozen in liquid nitrogen for histological analysis and RT-qPCR testing.

***Assessment of animal models***

The extensor posture thrust (EPT) was applied as a reflection of muscle strength of lower limbs in small animals (10). The rat was placed on the operating floor and the upper part of the body was grasped in a disinfect towels. Only the hindlimbs were exposed. One hindlimb was supported with the experimenter’s palm and the other hindlimb was placed upon a digital calculation balance. The flat surface of the scale is covered with a piece of rough absorbent paper. NEPT and EEPT represent extensor postural thrust on the normal and experimental sides. The formula for calculating the functional deficit ratio is: functional deficit ratio=(NEPT-EEPT)/NEPT.

The sciatic nerve function index (SFI) is the current standard for measuring functional recovery following rat sciatic nerve injury. The rat’s hindlimbs were dipped in a methylrosanilinium chloride solution, and it was permitted to walk down the track, which was covered with a strip of white paper. Measurements included print length on both the experimental and the normal sides (EPL, NPL), toe spread between the first and fifth digits on both sides (ETS, NTS), and the distance between the middle of the second and fourth toes on both sides (EIT, NIT). The formula for calculating the SFI is: SFI=109.5 (ETS-NTS)/NTS-38.3 (EPL-NPL)/NPL+13.3 (EIT-NIT)/NIT-8.8.

Consecutive gastrocnemius muscle sections were prepared in 5 μm thick sections. Histological examination of muscle fiber atrophy was assessed using hematoxylin and eosin (H&E) staining. The cross-sectional areas of the muscle fibers were measured with Image-Pro Plus, and at least 100 fibers per sample were counted (magnification×400).

***RT-qPCR***

Total RNA was extracted from the samples using TRNzol Reagent (DP405, TIANGEN) following the manufacturer’s protocol. Subsequently, the concentration (ng/ml) and purity of the total RNA were evaluated using the multimode reader. Only RNAs with OD_260_/OD_280_ ratios fell in 1.8-2.0 were used in subsequent experiments.

The first strand cDNA was synthesized with PrimeScript™ RT Master Mix (KB118, TIANGEN), following the manufacturer’s protocol. For the reverse transcription, 2 μg of total RNA was used in a final reaction volume of 20 μl.

Primers were designed and synthesized by Sangon Biotech (Shanghai)(Table 1). A 20 μl reaction mixture was prepared for real-time PCR amplification with SuperReal PreMix Plus (FP205, TIANGEN), containing 10 μl SuperReal PreMix Plus, 6.4 μl RNase-free ddH_2_O, 0.4 μl each of the forward and reverse primers of genes of interest and the reference gene, 0.4 μl 50×ROX Reference Dye, and 2.0 μl cDNA. The reaction was performed with an Applied Biosystems 7500, with one round predenaturation at 95 ^°^C for 15 min, and 40 rounds of denaturation at 95 ^°^C for 10 sec followed by annealing and extension at 60 ^°^C for 32 sec. Triple fluorescent signals (FAM, Cy5, and HEX or ROX) were measured synchronously at the end of each cycle. Fluorescence curves of the PCR products were evaluated using 7500 Software v2.0.5.

***Statistical analyses***

All results were expressed as mean±standard error (SE). The statistical difference was determined by one-way ANOVA. All statistical analyses were performed using SPSS, version 16.0, with *P-*value<0.05 considered statistically significant.

## Results

**Figure 1:**
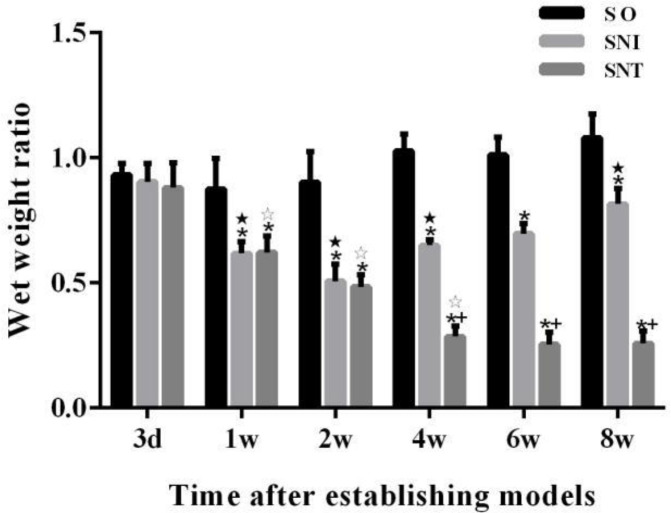
Gastrocnemius muscle wet weight ratio of rats

After the establishment of animal models, the gastrocnemius muscle atrophy of SNI and SNT groups was different from that of SO group. From week 4 to week 8, the gastrocnemius muscle atrophy of SNI group was significantly lighter than that of SNT group (*P-*value<0.05). The wet weight ratios of gastrocnemius in SNI and SNT groups decreased significantly in the first two weeks (*P-*value<0.05), but in the fourth week, the wet weight ratio of gastrocnemius in SNI group increased significantly compared to that in the second week (*P-*value<0.05), and then continued to show an upward trend. The wet weight ratio of gastrocnemius in SNT group did not change significantly from week 4 to week 8 (*P-*value>0.05) (Figure 1).

**Figure 2:**
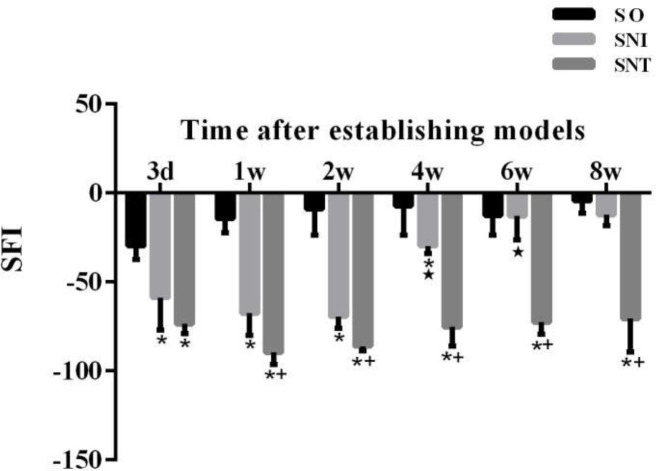
Sciatic nerve function index of rats

The SFI of SNI group was not significantly different from that of SO group from week 6 (*P-*value>0.05). And from week 2 to week 8, the sciatic nerve function of SNI group was less damaged than that of SNT group (*P-*value<0.05). From week 4, the sciatic nerve function of SNI group was better than that in the previous time point (*P-*value<0.05); while in SNT group, the sciatic nerve was always in a state of serious injury without significant change (*P-*value>0.05) (Figure 2).

**Figure 3:**
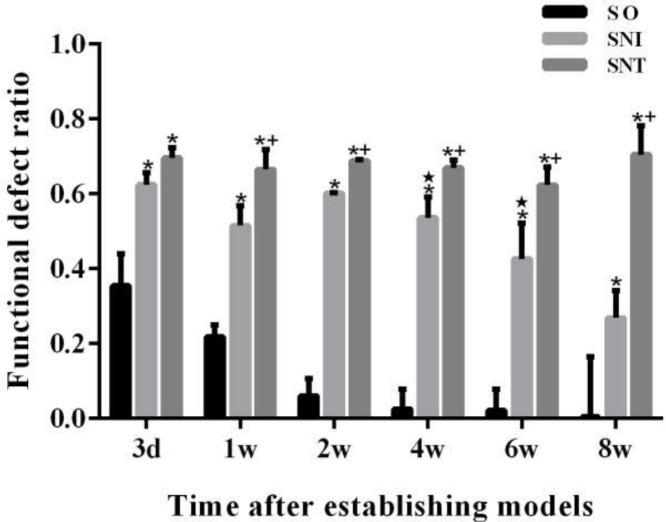
Functional defect ratio of rats

After the operation, the lower limb function of the operated side was impaired in all rats. The functional deficit ratios of SNI and SNT groups were significantly higher than that of SO group (*P-*value<0.05). The functional deficit ratio of SNT group was different from that of SNI group from week 1 to week 8 (*P-*value<0.05). In SNI group, the lower limb function of the operative side improved significantly compared to the previous time point from week 6 (*P-*value<0.05); in SNT group, the lower limb function of the operative side was severely impaired without change (*P-*value>0.05) (Figure 3).

**Figure 4:**
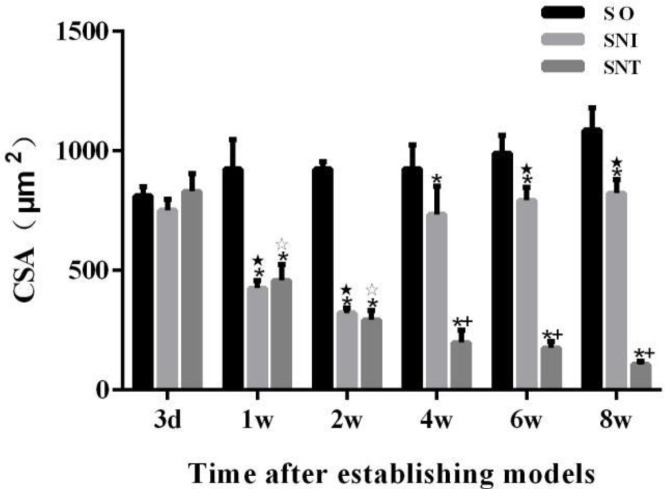
Gastrocnemius muscle fiber cross-sectional area of rats

**Figure 5.**
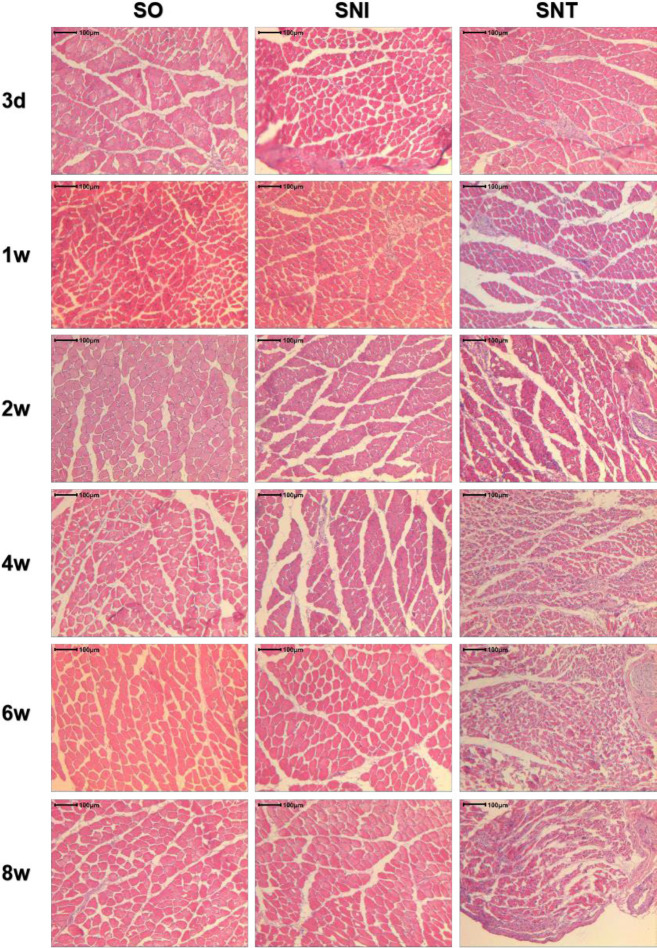
HE staining of gastrocnemius muscle cross section on the operative side of rats (100 times)

There was no significant change in the average area of gastrocnemius muscle fibers in each group on the 3^rd^ day after modeling (*P-*value>0.05). The cross-sectional areas of muscle fibers in SNI and SNT groups were smaller than that in SO group at the same time point from week 1 (*P-*value<0.05), but increased significantly in SNI group at the 4^th^ week (*P*<0.05). The cross-sectional area of muscle fibers in SNI group decreased in the first two weeks (*P-*value<0.05), but increased significantly at week 4 compared to the previous time point (*P-*value<0.05), while the cross-sectional area of muscle fibers in SNT group decreased continuously (Figure 4, 5).

**Figure 6:**
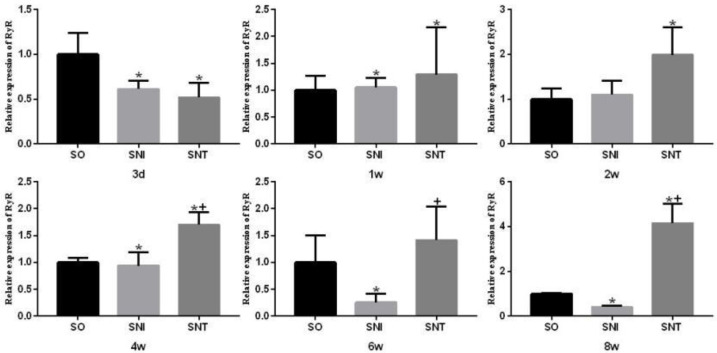
Transcription level of RyR1 in rat gastrocnemius muscle

The transcription level of RyR in SNI group was significantly higher than that in SO group at week 1 and decreased from week 4 to week 8 (*P-*value<0.05); the transcription level of RyR in SNT group was significantly higher than that in SO group at week 1, week 2, week 4 and week 8 (*P-*value<0.05) (Figure 6).

**Figure 7:**
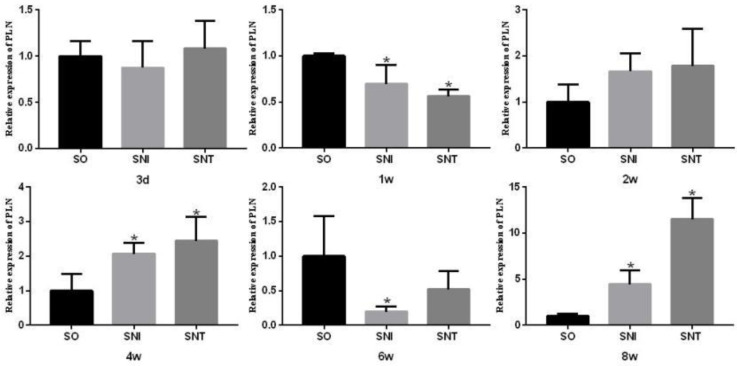
Transcription level of PLN in rat gastrocnemius muscle

Compared to SO group, the transcription levels of PLN in SNI and SNT groups decreased significantly at week 1 and week 6 (*P-*value<0.05), and increased at week 2, 4 and 8, especially at week 4 and week 8 (*P-*value<0.05) (Figure 7).

**Figure 8:**
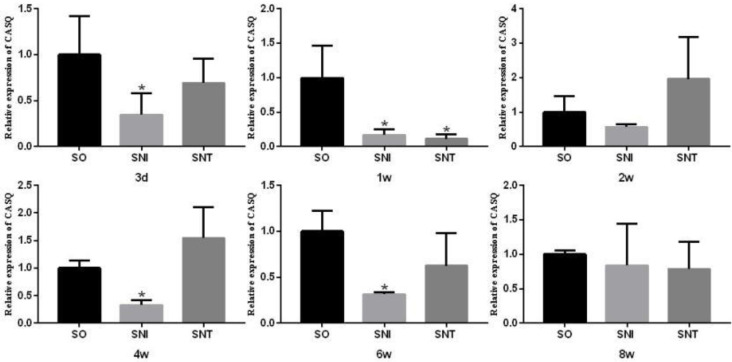
Transcription level of CASQ in rat gastrocnemius muscle

The transcription level of CASQ in SNI group was lower than that in SO group, which was more significant on the 3^rd^, 1^st^, 4^th^ and 6^th^ weeks (*P-*value<0.05). And that in SNT group was significantly lower than that in SO group only at the first week (*P-*value<0.05). There was no significant difference between SO group and SNT group at the other time points (*P-*value>0.05) (Figure 8).

**Figure 9:**
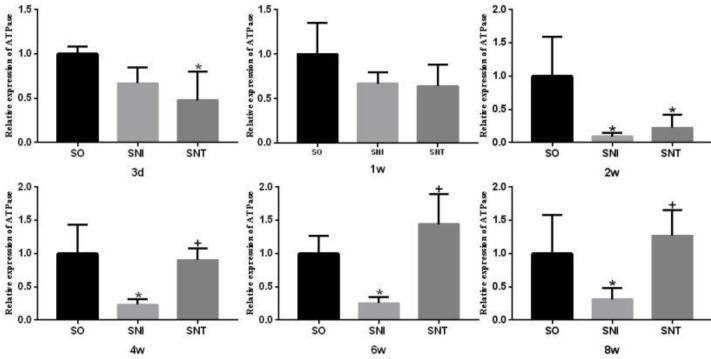
Transcription level of ATPase in rat gastrocnemius muscle

The transcription level of ATPase in SNI group was lower than that in SO group at week 2 (*P-*value<0.05); the transcription level of ATPase in SNT group was lower than that in SO group at the third day and the second week (*P-*value<0.05), and there was no significant change at other time points (*P-*value>0.05) (Figure 9).

**Figure 10:**
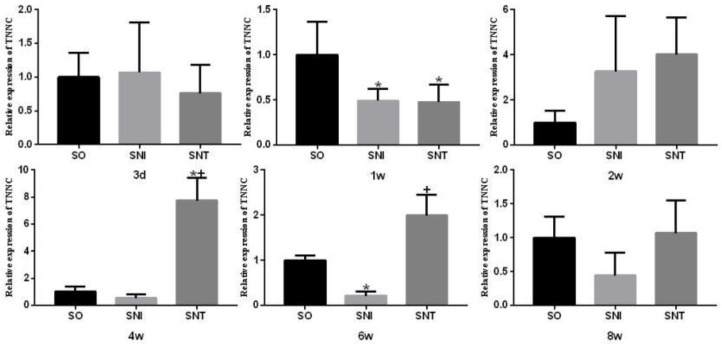
Transcription level of TNNC in rat gastrocnemius muscle

The transcription level of TNNC in SNI group was lower than that in SO group at week 1 and 6 (*P*<0.05), while that was lower at week 1 in SNT group, but significantly higher than that in SO group at week 4 (*P*<0.05) (Figure 10).

**Figure 11:**
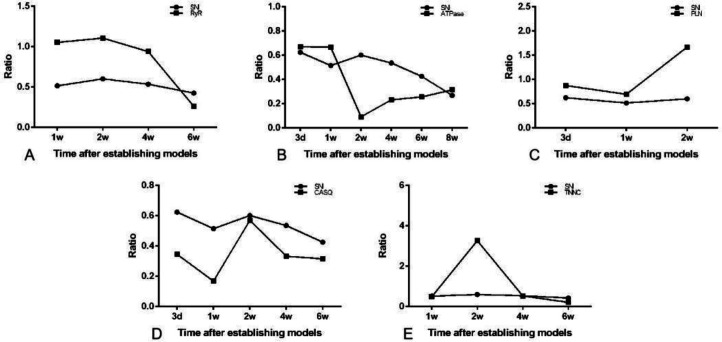
The transcription level of calcium cycle related genes in rat gastrocnemius muscle and the lower limb muscle function ratio in SNI group

The changes of transcription level of RyR and TNNC were opposite to those of muscle strength from week 1 to week 6; the changes of transcription level of PLN were opposite to those of muscle strength from day 3 to week 2, and the changes of transcription level of CASQ were opposite to those of muscle strength from day 3 to week 6. Also, the changes of transcription level of ATPase were the same as that of muscle strength throughout the experiment (Figure 11).

## Discussion

At present, identification of limb muscle strength in forensic clinical work still relies mainly on unarmed muscle strength identification method, which has the shortcomings of over subjective and poor repeatability. Therefore, we hope to find target genes that are related to the muscle strength changes caused by nerve injury muscular atrophy, and to explore an objective and reproducible method for muscle strength measurement.

The basic structural unit of skeletal muscle is muscle fiber, which is a slender multi-nucleated mechanism formed by the fusion of myoblasts. Skeletal muscle fibers are mainly divided into slow muscle fibers consisting of type I fibers and fast muscle fibers consisting of type IIA, type IIX and type IIB fibers. In addition, there are a few intermediate phenotypes of different fiber types due to fiber mixing. Rat’s gastrocnemius muscle is composed of slow and fast muscle fibers, and the proportion of the two kinds of muscle fibers is basically the same (11). The contractility of skeletal muscle is also closely related to the type of muscle fibers (12). After denervation, atrophic skeletal muscles exhibit malnutrition, accompanied by changes in fibers (13). In skeletal muscular atrophy, skeletal muscle fibers will gradually transform from type I to type II. Researchers have studied this phenomenon and found that type II muscle fibers are more likely to be dominated during the reinnervation of peripheral nerves after denervation, which may be a reason for muscle fiber conversion. It has been reported that the loss of muscle strength caused by nerve injury is greater than that observed in muscle atrophy (14). We hope to find a way to objectively express the changes of muscle strength in neuropathic muscular atrophy through this experiment. Therefore, we not only determine the degree of muscle atrophy through morphological experiments but also detect the changes of muscle strength of lower limbs in rats while maintaining a unified standard.

In this experiment, we found that the muscle loss in the SNI group was more severe than the muscle atrophy. This may be because the generation of muscle strength involves a variety of complex and coordinated biological processes (15) and does not depend entirely on muscle mass. Moreover, the excitatory-contraction coupling of skeletal muscle causes muscle contraction through calcium-mediated nerve stimulation, a process that is crucial for muscle strength production in skeletal muscle (16). Therefore, we studied the related changes of calcium cycle-related factors in the SNI model.

RyR is located in the sarcoplasmic reticulum terminal pool. There are three subtypes of these receptors including RyR1 (skeletal muscle), RyR2 (myocardium) and RyR3 (widely distributed, mainly in the brain). RyR1 is distributed in type I and type II muscle fibers. However, the distribution density of RyR1 in type II muscle fibers is higher than that of type I muscle fibers (17). RyR is an important component of the excitatory-contraction coupling process (18). After nerve stimulation, calcium ions in the sarcoplasmic reticulum can be released by RyR1 (19), and damage to this mechanism will lead to muscle contraction disorders. It has been reported that the expression of RyR is related to the muscle state. The transcription level of RyR1 changes with the degree of muscle atrophy after denervation of skeletal muscle in rats. There is a significant increase in RyR1 mRNA expression in the soleus muscle composed of typical slow muscle fibers after long-term denervation (20). However, the results of RyR transcript levels obtained in experiments with other types of muscle atrophy leading to decreased muscle strength are not same. Findings show that RyR expression may decrease (21), or may not change depending on situation (22). In this experiment, after significant muscle atrophy in the rat model, the transcriptional level of RyR1, which expressed superiority in fast muscle fibers, was significantly elevated. After muscle recovery in muscle atrophy rats, fast muscle fibers can be converted into slow muscle fibers (23). When the muscle mass of the SNI group recovered, the transcription level of RyR1 was significantly decreased, which may be related to this phenomenon. When the muscle strength of rats in the SNI group changed significantly in the late stage of the experiment, the transcription level of RyR1 was different from that of the other two groups of rats whose muscle strength was intact and muscle strength was lost. From week 1 to week 6, the trend of RyR1 transcription was contrary to that of muscle strength, which might be used for the muscle strength test at the molecular level.

PLN is a 52-amino-acid integral membrane phosphoprotein, which regulates the intracellular calcium concentration by regulating the activity of Ca^2+^-ATPase on the sarcoplasmic reticulum. Phosphorylated PLN increases the uptake of calcium ions in the sarcoplasmic reticulum and enhances the diastolic function of the muscle. PLN is present on the sarcoplasmic reticulum of the myocardium, slow contractile muscles and rapidly contracting muscles. Ca^2+^-ATPase on the sarcoplasmic reticulum is also known as sarcoplasmic reticulum Ca^2+^-ATPase (SERCA). SERCA is a major regulator of free calcium ions in skeletal muscle cells. It can use the energy released by ATP hydrolysis to recover free calcium ions from the sarcoplasmic reticulum into the sarcoplasmic reticulum (24). In mammals, SERCA is mainly divided into three gene categories of SERCA1 (ATP2a1), SERCA2 (ATP2a2) and SERCA3 (ATP2a3), and can produce more than 10 subtypes (25). SERCA1 and SERCA2 subtypes are dominant in adult skeletal muscle; PLN and SERCA2 are expressed mainly in slow muscle fibers, and SECRCA1 is mainly expressed in fast muscle fibers (26, 27). PLN regulates SERCA1 and SERCA2 by interacting with sarcolipin (SLN) to reduce the affinity of calcium ions on the sarcoplasmic reticulum surface (28). Studies have shown that after denervation, calcium concentration of the skeletal muscle sarcoplasmic reticulum decreases. PLN expression is significantly increased, but SECRCA activity is halved (29). In the early stage of experiment, the relative gene transcription level of PLN was opposite to that of muscle strength. The transcription level of ATP2a1 in the whole experiment was the same as that of muscle strength. This change in expression helps us to discover changes in muscle strength.

CASQ is the main binding protein for sarcoplasmic reticulum regulation of calcium storage, and it is mainly divided into CASQ1 and CASQ2 in mammals (30). In skeletal muscle, both CASQ1 and CASQ2 are expressed during development. CASQ2 is also abundantly expressed in adult slow muscle fibers, but CASQ2 expressed in fast muscle fibers is gradually replaced by CASQ1 after birth, and finally CASQ1 becomes the only CASQ subtype expressed by fast muscle fibers (31). Active calcium transport is limited by the concentration of free calcium ions in the sarcoplasmic reticulum (32). Fast muscle fibers release and absorb more calcium ions than slow muscle fibers. CASQ1 makes the free calcium ion concentration relatively low for SERCA more effective in the inward movement of calcium ions, which is essential for the maintenance of normal physiological activity of fast muscle fibers (33). It has been found that changes in muscle fiber types have a significant effect on calcium regulation in skeletal muscle during early denervation. The expression of CASQ1 with calcium storage function is slightly decreased (34), which is consistent with the change of transcriptional level of CASQ1 found in our early experiments. From the 3^rd^ day to the 6^th^ week, the transcription level of CASQ1 was contrary to the trend of muscle strength change, and it is expected to be used to detect muscle strength change.

TNNC is a complex composed of three subunits, troponin C, troponin I and troponin T. It is an important regulator of skeletal muscle contraction (35). It is divided into TNNC1 expressed in slow muscle fibers, TNNC2 expressed in fast muscle fibers and TNNC3 expressed in myocardium (36). Studies have shown that calcium ion in sarcoplasmic reticulum increases after denervation (37), and denervated skeletal muscle may increase the affinity of contractile proteins to calcium ions by increasing the expression of TNNC (38). This is consistent with the changes in the transcriptional level of TNNC2 found in the later period of our experiment. During the experiment, the transcriptional level of TNNC2 was opposite to that of muscle strength of rats in the SNI group.

Muscle strength changes in the SNI group can be attributed to complex interactions between multiple factors, such as signal transmission between nerves and muscles (39), muscle state (40) and excitation contraction coupling (41). In this experiment, we found that five calcium cycle-related genes have obvious expression changes in the SNI group, and have different expressions under different muscle strength states, and the transcriptional level of the gene and the trend of muscle strength are related in a certain period of time. However, the transcription level of housekeeping genes varies at different developmental stages, cell types, and experimental conditions (42). The transcriptional level of housekeeping genes in skeletal muscle is also affected by the sciatic nerve injury (43). In this experiment, we screened the housekeeping genes such as GAPDH, β-actin, Tbp, rpl32 and rps18 as internal parameters. Finally, combined with experimental results and previous experimental studies, GAPDH and rps18 were selected as double internal reference genes. Though there is no optimal internal reference gene, the relative quantitative transcriptional level of the calcium cycle-related genes is still different at different muscle strength levels in this experiment. This suggests that we can establish a new detection system to determine muscle strength by quantitative analysis of the expression of different genes.

**Table 1:** Primer sequence used for RTPCR

Gene	Forward primer	Reverse primer
RyR1	AGAGGCTCCTGTACCAGCAG	AGGATGGAGATGCCCAGCTT
PLN	AAGCCAAGGCCTCCTAAAAG	TGATAGCCGAGCGAGTAAGG
CASQ1	GTCAATGTTACGGATGCGGAC	CTCCTTTTCTCCAGCGAGGG
ATP2a1	CCCGAGATGGGGAAGGTCTA	TGCAATGTTGGTACCCGAGA
TNNC2	CCGCATCTTTGACAGGAACG	CCCAGAAGCCCGGAAAATCT
IP_3_R1	GTATTCCACGAAAGCATTCTCC	CTTTTTGTTTCCCAAATCGCTG
GAPDH	GACATGCCGCCTGGAGAAAC	AGCCCAGGATGCCCTTTAGT
Rps18	AAGTTTCAGCACATCCTGCGAGTA	TTGGTGAGGTCAATGTCTGCTTTC

* vs SO group at the same time *P-value<*0.05.+vs SNI group at the same time *P-value<*0.05. vs SNI group at the previous time *P-value<*0.05. vs SNT group at the previous time *P-value<*0.05

SO: Sham-operated group, SNI: Sciatic nerve injury group, SNT: Sciatic nerve disconnection group

* vs SO group at the same time *P-value<*0.05. + vs SNI group at the same time *P-value<*0.05. vs SNI group at the previous time *P-value<*0.05. SO: Sham-operated group, SNI: Sciatic nerve injury group, SNT: Sciatic nerve disconnection group

* vs SO group at the same time *P-value<*0.05. + vs SNI group at the same time *P-value<*0.05. vs SNI group at the previous time *P-value<*0.05. SO: Sham-operated group, SNI: Sciatic nerve injury group, SNT: Sciatic nerve disconnection group

* vs SO group at the same time *P-value<*0.05.+vs SNI group at the same time *P-value<*0.05. vs SNI group at the previous time *P-value<*0.05. vs SNT group at the previous time *P-value<*0.05

SO: Sham-operated group, SNI: Sciatic nerve injury group, SNT: Sciatic nerve disconnection group

* vs SO group *P-value<*0.05. + vs SNI group *P-value<*0.05

SO: Sham-operated group, SNI: Sciatic nerve injury group, SNT: Sciatic nerve disconnection group, RyR: Ryanodine receptor

* vs SO group *P-value<*0.05

SO: Sham-operated group, SNI: Sciatic nerve injury group, SNT: Sciatic nerve disconnection group, PLN: Phospholamban

*vs SO group *P-value<*0.05. + vs SNI group *P-value<*0.05

SO: Sham-operated group, SNI: Sciatic nerve injury group, SNT: Sciatic nerve disconnection group, CASQ: Cryptocalcitonin

*vs SO group *P-value<*0.05. + vs SNI group *P-value<*0.05

SO: Sham-operated group, SNI: Sciatic nerve injury group, SNT: Sciatic nerve disconnection group

*vs SO group *P-value<*0.05. + vs SNI group *P-value<*0.05

SO: Sham-operated group, SNI: Sciatic nerve injury group, SNT: Sciatic nerve disconnection group, TNNC: Troponin C

SNI: Sciatic nerve injury group

## Conclusion

The transcriptional level of calcium circulation-related genes, such as RyR1, PLN, CASQ and TNNC, changed with the time elapse after nerve injury in rat. They could be potential useful molecular markers for precise assessment of muscle strength after nerve injury applied in forensic clinical work.
